# Gastrointestinal and Respiratory Illness in Children That Do and Do Not Attend Child Day Care Centers: A Cost-of-Illness Study

**DOI:** 10.1371/journal.pone.0104940

**Published:** 2014-08-20

**Authors:** Remko Enserink, Anna Lugnér, Anita Suijkerbuijk, Patricia Bruijning-Verhagen, Henriette A. Smit, Wilfrid van Pelt

**Affiliations:** 1 Center for Infectious Disease Control, National Institute for Public Health and the Environment (RIVM), Bilthoven, The Netherlands; 2 Julius Center for Health Sciences and Primary Care, University Medical Center Utrecht, Utrecht, The Netherlands; 3 Center for Nutrition, Prevention and Health Services, National Institute for Public Health and the Environment (RIVM), Bilthoven, The Netherlands; University of British Columbia, Canada

## Abstract

**Background:**

Gastrointestinal and respiratory diseases are major causes of morbidity for young children, particularly for those children attending child day care centers (DCCs). Although both diseases are presumed to cause considerable societal costs for care and treatment of illness, the extent of these costs, and the difference of these costs between children that do and do not attend such centers, is largely unknown.

**Objective:**

Estimate the societal costs for care and treatment of episodes of gastroenteritis (GE) and influenza-like illness (ILI) experienced by Dutch children that attend a DCC, compared to children that do not attend a DCC.

**Methods:**

A web-based monthly survey was conducted among households with children aged 0–48 months from October 2012 to October 2013. Households filled-in a questionnaire on the incidence of GE and ILI episodes experienced by their child during the past 4 weeks, on the costs related to care and treatment of these episodes, and on DCC arrangements. Costs and incidence were adjusted for socioeconomic characteristics including education level, nationality and monthly income of parents, number of children in the household, gender and age of the child and month of survey conduct.

**Results:**

Children attending a DCC experienced higher rates of GE (aIRR 1.4 [95%CI: 1.2–1.9]) and ILI (aIRR: 1.4 [95%CI: 1.2–1.6]) compared to children not attending a DCC. The societal costs for care and treatment of an episode of GE and ILI experienced by a DCC-attending child were estimated at €215.45 [€115.69–€315.02] and €196.32 [€161.58–€232.74] respectively, twice as high as for a non-DCC-attending child. The DCC-attributable economic burden of GE and ILI for the Netherlands was estimated at €25 million and €72 million per year.

**Conclusions:**

Although children attending a DCC experience only slightly higher rates of GE and ILI compared to children not attending a DCC, the costs involved per episode are substantially higher.

## Introduction

Over the last decades, the number of children attending day care centers (DCCs) has increased all over the world [1]–[4] including the Netherlands [5], reflecting the desire and need of parents to provide for family income while their children are cared for in a safe environment. Quinquagintupled since 1980 [5], half of the 0.7 million Dutch children aged 0–48 months spends an average of 2.5 days in one of 6000 day care centers (DCCs) active in the Netherlands today.

The shift from home-care to out-of-home care has had a significant impact on the attending child's health. Indeed, numerous studies convincingly demonstrate that children experience a 2–3 times increased risk of mild/moderate [6]–[20] and severe [21], [22] gastrointestinal and respiratory disease episodes around the time these children start attending center-based care. A question less frequently raised is whether the excess DCC-attributable risk also translates into excess societal costs. Attending a DCC might well shift the occurrence of first infectious disease episodes to an earlier age where complications requiring health care, and thus costs, are more likely to arise. Indeed, the impact of the increased infectious disease risk associated with the DCC setting seems not restricted to the clinical domain. Several studies have shown that DCC attendance may lead to substantial societal costs due to increased health care visits, medication usage and parental productivity losses as a result of a child's illness [23], [24].

In the Netherlands, both the extent of the societal costs per episode of gastrointestinal and respiratory disease experienced by young children, and whether these costs differ between children that do and do not attend a DCC, are largely unknown. Estimates of these costs, and the role of day care therein, would aid epidemiologists and health economists to assess the desirability and feasibility of intensified avocation of preventive measures in day care from a financial in addition to a clinical point of view. The aim of this study was therefore to (1); estimate the societal costs for care and treatment of a child 0–4 years old experiencing an episode of gastroenteritis (GE) and influenza-like-illness (ILI). (2); compare these costs between children that do and do not attend center-based day care.

## Material and Methods

### Ethics statement

The Dutch Central Committee on Research involving Human Subjects in Utrecht, The Netherlands, gave permission to conduct this study (protocol number: 13-051/C). Given that no subject-identifiable data were generated and the surveillance activities implied no risk or burden for any individuals, the committee judged that no specific ethical permission was required for individual consent. This study is conducted according to the principles of the Declaration of Helsinki.

### Design, definitions and study population

This study is part of a larger web-based, cross-sectional survey on the contribution of young children in transmission of infectious diseases to their families and vice versa [25]. Our study utilized data from this survey collected during October 2012-October 2013. Each month, 2000 children - one child per household – were randomly selected from the population registries of 415 Dutch municipalities. We selected only those households with children eligible for DCC attendance, aged 0–48 months old. Parents these households – one parent per household – were asked fill-out a standardized digital questionnaire on the occurrence of GE and ILI episodes experienced by their child during the past 4 weeks and on the costs related to care and treatment of these episodes.

Predefined respiratory symptoms and syndromes in the questionnaire included fever (sudden onset of fever (≥38°C) and/or warm to the touch with suspicion of fever), cough (sudden and frequent occurring tussis), rhinitis, sore throat, frontal headache, retrosternal pain and myalgia. Predefined gastrointestinal symptoms and syndromes included diarrhea (sudden, non-chronic, onset of >3 episodes of watery stools per day), vomiting (sudden, non-chronic, onset of >3 emetic episodes per day), abdominal pain, abdominal cramps, nausea, blood in the stool, mucus in the stool. Predefined symptoms and syndromes were used to post-define the case definitions of GE and ILI. Consequently, GE was defined as three or more loose stools per day or diarrhea during at least one day accompanied by ≥2 additional symptoms, or vomiting with ≥2 additional symptoms during the past four weeks [26], [27]. Additional symptoms included abdominal pain, abdominal cramps, nausea, blood in the stool, mucus in the stool, diarrhea and vomiting. ILI was defined as an acute or sudden onset of symptoms with fever (≥38°C) and ≥1 of the following symptoms: cough, rhinitis, sore throat, frontal headache, retrosternal pain and myalgia [27]. The case definitions were very similar to those used in previous day care- [28] community- [29] and GP-based [28], [30] studies.

In addition, the questionnaire included inquiries about the parent's about utilized health care resources and absenteeism from work for care and treatment of illness experienced by their child. In addition, parents were asked to provide information on the demographic and socioeconomic characteristics of their household, including household income, number of members, work status of parents, DCC arrangements and hygiene habits. Each question required an answer before a parent could proceed to the next question. The questionnaire was sent to us only upon completion of the last question.

### Economic parameters

Per GE and ILI episode, we considered three types of costs related to the use of resources for care and treatment of illness: (1) Direct health care costs (DHC); costs related to doctor consultation, (over-the-counter) medication, hospitalization and laboratory testing. (2) Direct non-health care costs (DNHC); costs related to traveling to and from health care services. (3) Indirect non-health care costs (INHC); costs related to productivity losses due to absence from work of parents to care for their ill child. The costs per resource unit per disease episode were calculated as the number of resource units utilized (number of GP visits, number of productivity days lost etc.) multiplied by the costs per resource unit. Together, the three types of costs determined the societal costs of an illness episode [31]–[34]. An overview of the categories, resources units and unit costs is provided in table 1. All three categories, as well as the unit costs of resources belonging to these categories, are in accordance with the Dutch guidelines for health economic evaluations [35] which formed the basis for.

**Table 1:** Unit costs in the Netherlands, 2013 (all costs are in Euros).

Resource unit	Unit cost (€)	Ref.
***Direct health care costs***		
Doctor (per visit)*	28	[35]
Medication (including prescription charges)**	9	[35]
Laboratory testing (per request)***	13	[35]
Hospital admission children (per day)	615.75	[35]
***Direct non-health care costs***		
Car/public transport (per km)****	0.21	[35]
Parking fees (per visit)	3.11	[35]
***Indirect non-health care costs***		
Productivity loss due to absence from paid work (per hour)*****		
15–19 years	9.61	[35]
20–24 years	18.15	[35]
25–29 years	24.80	[35]
30–34 years	29.85	[35]
35–39 years	33.43	[35]
40–44 years	35.16	[35]
45–49 years	36.14	[35]
50–54 years	36.91	[35]
55–59 years	37.70	[35]
60–64 years	37.74	[35]

*We regarded all consultations as though children had visited the doctor.

**The costs of antibiotics, antiviral and other medications prescribed by a GP were assumed equal and included pharmaceutical fees.

***Costs for analyzing blood, urine, respiratory and fecal material in the laboratory were assumed equal.

****We assumed that transport to a doctor would cost €0.21 per km regardless whether care or public transport was used. We set the average distance from a household to a doctor at 1.1 km.

*****Productivity losses were estimated per working days lost (1 day = 8 hours' work) using standard tariffs according to gender and age-class.

### Assumptions

The following assumptions were made for travelling costs and productivity losses due to absenteeism from paid work based on previous published work [34]: we only considered direct non-health care (travelling) costs if a child visited a doctor in a general practice or in a hospital. We assumed that over-the-counter medicines would not lead to additional traveling costs. We set the average distance from a household to a doctor at 1.1 km. Productivity losses were estimated per working days lost (1 working day = 6.3 hours) using standard tariffs according to gender and age-class. We regarded all consultations as if children had visited the doctor.

### Statistical analyses

#### Estimating GE and ILI incidence

The average yearly incidence rate of GE and ILI was calculated as (365/30) x (average four-weekly incidence rate) x 1000 children, since the period of observation was 1 month and we want to express the incidence rate per 1000 child-years. The child-time at risk, the incidence denominator, was defined as the number of participating households per month.

#### Estimating the mean societal cost per GE and ILI episode

The costs due to illness represent a semi-continuous outcome. They are characterized by a point mass at zero (representing illness episodes for which no costs were made), followed by a right-skewed continuous distribution of positive values (representing illness episodes for which costs were made). We therefore applied a two-part regression model consisting of a logistic and a log-linear component using the Stata module tpm for cross-sectional models which are commonly used in cost of illness studies [36], [37]. Several previously conducted cost-of-illness studies [38], [39] and burden studies [40] in the Netherlands. All costs were indexed to Euros (€) 2012. No discounting needed to be performed given the one year study period [35]. In the logistic component, we estimated the probability that there were any costs on resources for care and treatment of illness. In the log-linear component, we estimated the size of the log-transformed costs of a household, given that the household made costs for care and treatment of disease experienced by their child. The societal costs were estimated by multiplying the probability of making costs (logistic component model) by the estimated costs per illness episode (log-linear component model). The estimated log costs were backcalculated to Euros. Both incidence and societal costs estimates were adjusted for the socioeconomic status and urbanization degree of the household neighborhood, the education level and monthly income of parents, the number of additional children in the household, the nationality, gender and age of the selected child and the month of survey conduct. Socioeconomic status was expressed as “high socio-economic status” and “low socio-economic status based on the level of income, employment and educational level per postal code area of the household. The urbanization degree was expressed as “urbanized” (1500–2.500 addresses/km^2^) and “rural” (0–1500 addresses/km^2^). The education level was ‘high’ if one or both parents had a university diploma, and low otherwise. The number of additional children was categorized as 0, 1,and >1. Children were considered “Dutch” if both parents were born in the Netherlands), “Other Western” if one of the parents was born in a European country other than the Netherlands, and “Other” in all other instances. The age of the child was expressed as above or equal to/below 24 months of age.

The mean estimated adjusted costs per GE and ILI episode were estimated only for the total societal costs. For these costs, bootstrap analyses with replacement (1000 simulations per run) were conducted to estimate the sampling distributions of estimated adjusted mean costs, providing 95% confidence intervals around cost estimates. For the separate resource unit costs and the DHC, DNHC and INHC subtotals, the mean crude costs were provided. These were calculated by multiplying the mean frequency of resource usage per episode times the costs per resource unit. Separate analyses were performed for households with and without DCC-attending children. The annual costs for the Dutch community were estimated as the mean adjusted costs per illness episode times the excess day-care associated incidence per 1000 child-years times the population size of children aged 0–4 year old in the Netherlands attending DCCs. We used STATA/SE 12.0 StataCorp LP, USA) for all descriptive and multivariate analysis.

#### Correction for non-response

We applied inverse probability or survey weighting to correct both the disease incidence and cost estimates per episode of GE and ILI for underreporting of households based on their socioeconomic status, urbanization degree and age of the child. This allowed us to extrapolate the incidence and costs estimates to the Dutch community in 2012. Socioeconomic characteristics of the household were derived from the population registries from which households were selected, dichotomized and consequently assigned to each participating households. Inverse probability weighting is widely used in household surveys to correct for the potential biasing impact of nonresponse.

## Results

### Respondents vs non-respondents, DCC vs no DCC

**Figure 1:**
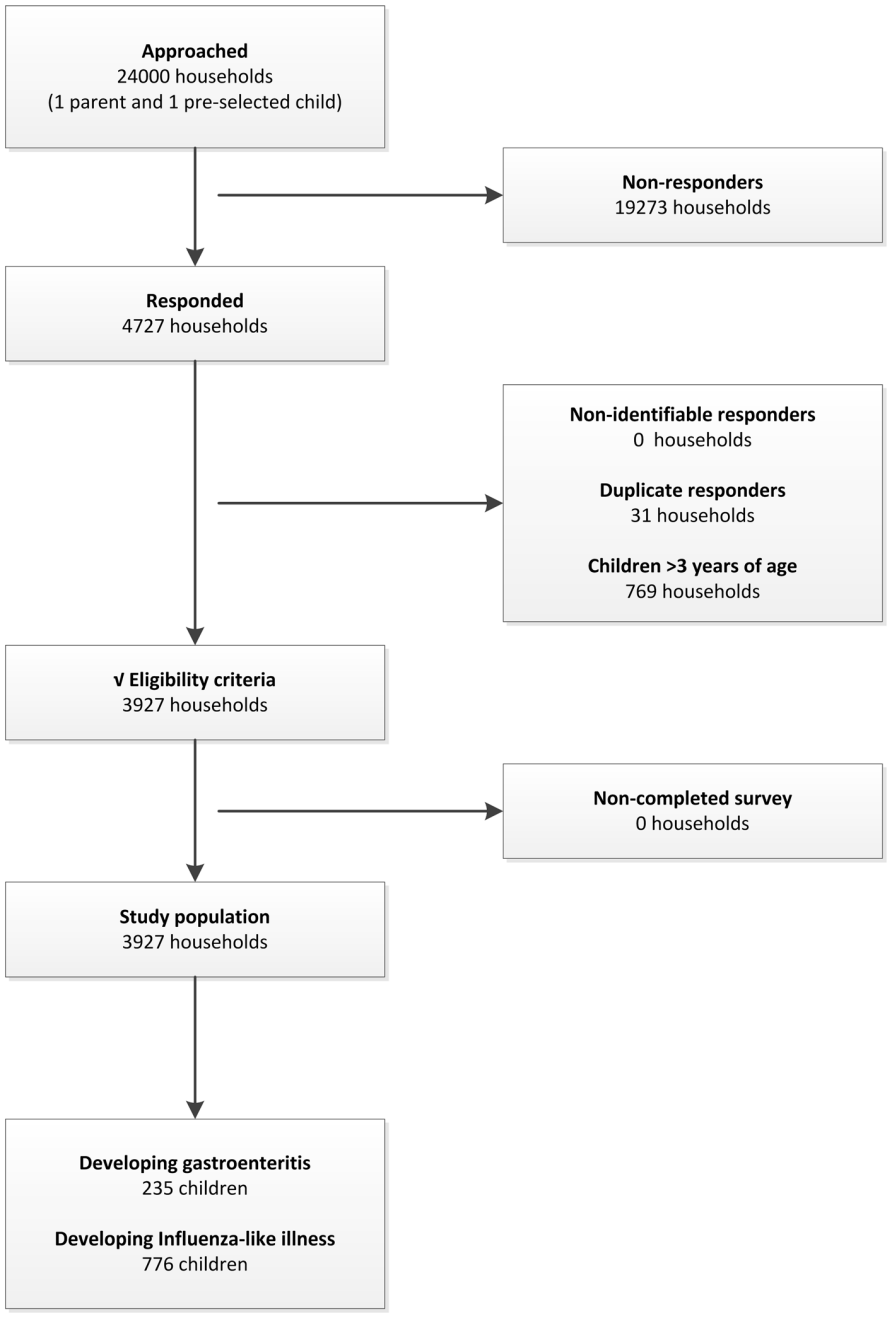
Number of approached, responding and analyzed households, including number of children developing GE or ILI during the study period October 2012–October 2013.

Of the 24.000 households approached during the study period, 4727 responded to the survey and 3927 met the eligibility criteria (figure 1). Compared to non-respondents, respondents were slightly more likely to live in urbanized areas with a higher socio-economic status and more often were of the Dutch nationality (table 2). Respondents and non-respondents did not differ with respect to the age of the child, nor the child's DCC-attendance status. Compared to children that did not attend a DCC, children that did were more likely to come from a household with few siblings (<2), well-educated and employed parents (university degree, both parents employed), and with a high income (>€3600/month, table 2). Given these differences, we ensured that households with and without DCC-attending children were identical in terms of socio-economic status and urbanization degree when estimating disease incidence and related costs for care and treatment of disease.

**Table 2:** Socio-demographics of households that did (n = 4727) and did not respond (n = 19273) to our questionnaire survey and (respondents) for households that have (n = 1930) and do not have a child (n = 1997) attending a DCC.

	N = 4727 respondents Characteristics			Ratio [95% CI] Reference: no day care
Response Yes/No				
		**Respondent (n = 4727)**	**Non-respondent (n = 19273)**	
	**Neighborhood**			
	High urbanization degree^1^, % (n)	52.4	46.8	1.12 [1.09–1.15]
	High socio-economic status^2^, % (n)	58.2	52.0	1.12 [1.09–1.15]
				
	**Child**			
	Age in months, mean (n)	24.3	24.6	0.96 [0.91–1.01]
	Attending DCC, % (n)	49	47^1^	1.04 [0.98–1.09]
	Dutch nationality, % (n)	96.7	80^1^	-
DCC Yes/No				
		**DCC (n = 1930)**	**No DCC (n = 1997)**	
	**Neighborhood**			
	High urbanization degree^2^, % (n)	56.3	48.0	0.85 [0.80–0.90]
	High socio-economic status^3^, % (n)	63.6	53.0	1.18 [1.10–1.27]
				
	**Child**			
	Age in months, mean (n)	25.5	22.9	1.11 [0.99–1.14]
				
	**Respondent**			
	Gender male, % (n)	18.0	18.7	1.03 [0.90–1.18]
	Dutch Nationality, % (n)	97.1	96.3	0.99 [0.90–1.08]
	Both parents employed, % (n)	94.5	73.9	1.24 [1.24–1.32]
	Working hours/month, mean (n)	26.5	18.8	1.40 [1.39–1.42]
				
	**Household**			
	High household income >€3600/month, % (n)	39.3	15.4	2.56 [2.28–2.88]
	University diploma in household, % (n)	73.4	51.6	1.42 [1.35–1.50]
	No of children living in household, mean (n)	1.8	1.9	0.93 [0.89–0.97]

1 Based on a population data estimates from the Central Bureau of Statistics, the Netherlands.

2 Addresses/km^2^. An urbanized neighborhood was defined as 1500–2.500 addresses/km^2^.

3 Normalized score (−4–4) based on level of income, employment and educational level per postal code area of the neighborhood. A high socio-economic status was defined between −4 and 0.

### Disease incidence of GE and ILI

Table 3 provides the incidence rate estimates for GE and ILI episodes among children, stratified by the child's age in years and by DCC attendance. During the study period, parents reported 235 episodes of GE and 776 episodes of ILI among their children during 3927 child-months of observation. The estimated mean incidence of GE and ILI for 0–4 year children attending a DCC was 1251 [95% confidence interval (CI): 1028–1476] per 1000 child-years and 3672 [95% CI: 3267–4007] per 1000 child-years respectively. For non-day-care-attending children, these incidence estimates were 893 [95% CI: 698–1089] and 2538 [95% CI: 2701–3407]. Thus, children that attended a DCC experienced a slightly significant higher rate of GE (adjusted incidence rate ratio (aIRR): 1.4 [95% CI: 1.2–1.9], mean excess incidence: 358 episodes per 1000 child-years) and ILI (IRR: 1.4 [95% CI: 1.2–1.6], excess incidence: 1134 episodes per 1000 child-years) compared to non-DCC-attending children. Subgroup analyses per year of age demonstrated that the rate differences for GE and ILI between DCC- and non-DCC-attending children was significantly more pronounced for children between 1 and 2 years of age. We could not reliably estimate the incidences for children below the age of one year given our low numbers in this age category.

**Table 3:** Incidence of gastroenteritis (GE) and Influenza-like Illness (ILI) among children that do and do not attend a DCC, stratified by the age of the child.

	DCC-attending child			Non-DCC-attending child			
Syndrome	Cases (n)	at risk (n)	Incidence rate (per 1000 child-years)	Cases (n)	at risk (n)	Incidence rate^1^ (per 1000 child-years)	Incidence rate ratio^2^ [95% CI]
**GE**							
0 years	5	60	1000 [154–1846]	2	124	194 [24–461]	5.2 [0.9–54.3]
1 years	37	463	959 [662–1256]	31	628	592 [389–796]	1.6 [1.1–2.7]
2 years	42	692	728 [515–942]	27	655	495 [312–677]	1.5 [.9–2.5]
3 years	51	715	856 [629–1082]	40	590	814 [570–1057]	1.1 [.7–1.6]
** Crude**	**135**	**1930**	**839 [703–976]**	**100**	**1997**	**601 [486–716]**	**1.4 [1.1–1.8]**
** Adjusted** 1	**135**	**1930**	**1251 [1028–1476]**	**100**	**1997**	**893 [698–1089]**	**1.4 [1.2–1.9]**
**ILI**							
0 years	7	60	1400 [417–2383]	6	124	581 [126–1036]	2.4 [0.7–8.7]
1 years	137	463	3551 [3051–4050]	129	628	2465 [2085–2844]	1.4 [1.1–1.9]
2 years	137	692	2376 [2019–2732]	121	655	2217 [1860–2574]	1.1 [0.8–1.4]
3 years	136	715	2283 [1937–2628]	102	590	2095 [1727–2463]	1.1 [0.8–1.4]
** Crude**	**417**	**1930**	**2593 [2372–2813]**	**359**	**1997**	**2157 [1955–2359]**	**1.2 [1.1–1.4]**
** Adjusted** 1	**417**	**1930**	**3672 [3267–4007]**	**359**	**1997**	**2538 [2701–3407]**	**1.4 [1.2–1.6]**

1 Incidence rates are adjusted for the socioeconomic status and urbanization degree of the household neighborhood, the education level and monthly income of parents, the number of children in the household, the nationality, gender and age of the selected child and finally, the month of survey conduct.

2 Ratio between disease incidence among households with and without (reference) DCC-attending children.

### Societal costs of an episode of GE and ILI

Table 4 presents the resources used and societal costs estimated per episode of GE and ILI adjusted for the socio-economic status and degree of urbanization of the household. Parents reported 122 episodes of GE and 84 episodes of ILI among their children for which societal costs were made. The adjusted mean costs per episode of GE and ILI were estimated at €215.45 [€115.69–€315.02] and €196.32 [€161.58–€232.74] for a child attending a DCC respectively. For a child not attending a DCC, these costs were estimated at €90.56 [€45.32–€135.75] and €95.20 [61.48–127.38] per illness episode respectively. Productivity losses accounted for the majority of the differences in mean costs between children attending and not attending a DCC. Both households with and without children attending a DCC experienced approximately one and a half days of work days lost per episode of GE and ILI if productivity losses were involved. However, the probability of experiencing productivity losses, was higher for households utilizing DCC services.

**Table 4:** Crude and adjusted mean costs for resources utilized per episode of gastroenteritis (GE) and Influenza-like Illness (ILI).

			GE (N = 95)				ILI (N = 297)		
			DCC+		DCC-		DCC+		DCC-
	Resource	Cases^1^ n (freq^2^)	Costs mean €	Cases n (freq)	Costs mean €	Cases n (freq)	Costs mean €	Cases n (freq)	Costs (mean €)
**DHC**									
	Doctor	41 (65)	16.51 [11.09–21.92]	41 (67)	20.36 [14.04–26.69]	31 (48)	17.17 [11.38–22.96]	31 (51)	26.96 [18.18–35.74]
	Medication	22 (29)	1.81 [1.06–2.56]	25 (30)	2.45 [1.57–3.34]	32 (44)	5.04 [3.55–6.53]	27 (37)	6.5 [4.51–8.49]
	Laboratory	8 (8)	0.79 [0.25–1.32]	8 (8)	1.07 [.35–1.79]	5 (5)	0.88 [.12–1.64]	2 (2)	0.49 [.2–1.17]
	Hospital	5 (6)	22.99 [2.96–43.01]	1 (1)	6.22 [−6.13–18.57]	2 (2)	16.43 [−6.56–39.41]	1 (1)	11.41 [−11.47–34.29]
	**Crude subtotal**	**47**	**42.09 [18.62–65.57]**	**46**	**30.10 [15.26–44.95]**	**43**	**39.52 [14.26–64.77]**	**37**	**45.36 [18.03–72.68]**
**DNHC**									
	Transport^3^	41 (65)	2.21 [1.42–3.00]	41 (61)	2.48 [1.68–3.29]	31 (50)	2.15 [1.38–2.92]	31 (51)	3.26 [2.11–4.41]
	**Crude subtotal**	**41**	**2.21 [1.42–3.00]**	**41**	**2.48 [1.68–3.29]**	**31**	**2.15 [1.38–2.92]**	**31**	**3.26 [2.11–4.41]**
**INHC**									
	Productivity loss parent	32 (51)	109.62 [66.34–152.90]	16 (22)	56.00 [27.5–84.5]	16 (25)	112.38 [50.47–174.3]	8 (14)	49.84 [14.39–85.29]
	Productivity loss partner	25 (33)	75.29 [43.12–107.46]	11 (10)	33.22 [12.88–53.56]	9 (12)	60.40 [17.47–103.33]	4 (5)	19.77 [.51–39.02]
	**Crude subtotal**	**42**	**184.91 [122.57–247.26]**	**21**	**89.22 [50.62–127.82]**	**21**	**172.78 [88.71–256.85]**	**9**	**69.61 [22.47–116.74]**
**TOTAL**									
	**Crude total**	**68**	**229.25 [152.17–306.34]**	**54**	**121.80 [79.19–164.51]**	**46**	**214.45 [115.14–313.75]**	**38**	**118.23 [64.78–171.67]**
	**Adjusted total** 4	**68**	**215.45 [115.69–315.02]**	**54**	**90.56 [9.90–171.22]**	**46**	**196.32 [161.58–232.74]**	**38**	**95.20 [61.48–127.38]**

1 Number of children that required on or more resources per episode of illness.

2 Number of visits to the general practitioner, number of medication prescriptions, the number of laboratory tests, number of days hospitalized, number of transports, number of work days lost.

3 Includes parking fees.

4 Calculated using two-part regression models. Cost estimates are adjusted for the socioeconomic status and urbanization degree of the household neighborhood, the education level and monthly income of parents, the number of children in the household, the nationality, gender and age of the child and the month of survey conduct.

Costs are stratified on households with (DCC+) and without (DCC-) a day-care-attending child.

### Yearly costs for the Dutch community

In 2012, there were approximately 700.000 children aged 0–48 months eligible to attend center-care in the Netherlands. Of these children, 324.800 (46.4%) were registered for DCC attendance. Using the adjusted excess disease incidence associated with DCC attendance, the adjusted mean costs per disease episode, and the number of children registered for DCC services, we estimated the additional costs related to care and treatment of GE and ILI episodes standardized for the Dutch community in 2012. The additional community costs in 2012 for GE and ILI episodes related to DCC attendance were estimated at €25 million and €72 million, respectively.

## Discussion

Using a unique general-population-based approach, our study has quantified the (differences in) GE and ILI incidence and related health care costs and productivity losses among children that do and do not attend a DCC in the Netherlands during October 2012–October 2013. Compared to home-cared children, DCC-attending children experience a slightly higher incidence of gastroenteritis (GE) and influenza-like illness (ILI). Yet the societal costs for care and treatment of an episode of GE and ILI are substantially higher for children attending DCCs. The cost differences are predominantly caused by the higher productivity losses experienced by parents with DCC-attending children for care and treatment of their ill child. Our results suggest that a small reduction in the excess number of disease episodes associated with DCC attendance could lead to a substantial reduction in the societal costs these episodes incur.

The GE incidence rates presented in this study are comparable with findings from a Dutch population-based cohort study on the incidence of GE in the Netherlands performed in 1998 [26]. This study estimated the GE incidence at 900 episodes per 1000 child-years [95% CI: 766–2034] for 0–4 year old children, which approximates our estimation of 1251 per 1000 child-years [95% CI: 1028–1476] for GE in this age group. The societal costs estimates for an episode ILI were comparable with findings from a prospective DCC cohort study performed in Australia in 2010 [24]. This study estimated the median societal cost of an ILI episode for a DCC-attending child at AU$321 (€180) compared to €196 in this study.

Some general remarks are in place to put the estimates presented in this paper in perspective. (1) Although all incidence and cost estimates were corrected for non-response and were adjusted for several socioeconomic factors, the low response rate coupled with a slightly higher participation rate among children from households with a higher socioeconomic background and Dutch nationality might limit the generalizability of our findings. (2) Rates of opting-out, if any, might have differed between households with and without DCC-attending children for those questions that households refused or considered irrelevant to answer - but needed to answer - before being able to proceed to the next question. (3) The societal costs were calculated based on standardized unit costs rather than actual costs, reasoning that doing so would increase the likelihood of parents filling-in the complete questionnaire. This approach naturally assumes that identical costs apply for households of which the child does or does not attend a DCC, when in fact these costs may differ. For example, attending a DCC might translate into episodes of GE and ILI that are not only greater in number, but also in severity [20]–[22]. If such differences in severity manifested themselves in costs not captured by our survey, we might have underestimated the societal costs for children attending DCCs. Another consequence of the compromise between precision and logistic feasibility of our survey is that we did not include all possible resource units and confounders. For instance, we did not consider food consumption of parents in the hospital if their child was admitted, nor the costs associated with the increased consumption of e.g. diapers during illness. Furthermore, our survey did not extend to asking about the use of functional foods, although these have been associated with a reduction in the occurrence of acute gastroenteritis among young children [41]. (4) In the Netherlands, there are two major types of formal out-of-home care facilities: the DCC, or formal center-based care and the day care home, or formal home-based care [42]. Day care homes care for 1 to 6 children, whereas DCCs provide care for more than five and up to a few hundred children. In this manuscript, we focus solely on the DCCs. Our results may therefore not be generalizable to the smaller day care homes. One large and well-conducted study observed no difference in risk between day care homes and DCCs [22]. Yet several other studies suggest that the occurrence and costs associated with GE and ILI might differ according to the type of childcare facility attended, although controversy remains as to whether the highest risk is confined to the day care homes [43]–[45] or the DCC [9], [13], [15], [46], [47]. (5) We performed a cost-of-illness analysis, not a cost-benefit analysis. To assess the full significance of our cost estimates, future research needs to consider the potential cost-benefits of out-of-home child care services for the society as a whole. For instance, studies would need to take into account the economic contribution of parents that can join the labor force because their children are cared for at a DCC. The costs associated with illness are likely to be dwarfed by such economic contributions. In addition, the long-term costs associated with day care would need to be considered. Attending a DCC might well confer long-term beneficial effects on infectious disease morbidity, e.g. if early attendance is associated with repeated immune challenges posed by the higher levels of exposure to pathogens circulating in the day care environment [48]. Such effects have for example been reported in some studies for respiratory illness (although not gastrointestinal illness) which suggested that an excess of day care-related respiratory illness experienced by children early in life results in a reduction in the risk of developing allergic disease[49]–[53] and mild respiratory infections[54]–[57] in life. If true, the societal cost for children not attending DCCs might not be avoided, but only delayed until elementary school [12], [21], [56].

Concluding, this study provides indications for the overall economic scale of the community burden of GE and ILI associated with day care attendance in the Netherlands. Its main message is not that DCCs are costly and that we should refrain from sending children to day care. Rather, our results suggest that lowering the disease incidence of major childhood diseases in the DCC setting could potentially lead not only to a reduction of the DCC-related clinical impact, but also to considerable societal cost savings. As such, the incidence and cost estimates presented here are of concern not only to parents, employers and health insurers, but also to public health officials and DCC organizations involved in setting priorities and allocating resources for infection prevention in DCCs.
